# Behavioral Evidence and Olfactory Reception of a Single Alarm Pheromone Component in *Halyomorpha halys*

**DOI:** 10.3389/fphys.2018.01610

**Published:** 2018-11-13

**Authors:** Yong-Zhi Zhong, Rui Tang, Jin-Ping Zhang, Shi-Yong Yang, Guo-Hua Chen, Kang-Lai He, Zhen-Ying Wang, Feng Zhang

**Affiliations:** ^1^MARA-CABI Joint Laboratory for Bio-safety, Institute of Plant Protection, Chinese Academy of Agricultural Sciences, Beijing, China; ^2^College of Plant Protection, Yunnan Agricultural University, Kunming, China; ^3^State Key Laboratory of Integrated Management of Pest Insects and Rodents, Institute of Zoology, Chinese Academy of Sciences, Beijing, China; ^4^Centre for Agriculture and Biosciences International (CABI) East Asia, Beijing, China; ^5^Life Science College, Anhui Normal University, Wuhu, China

**Keywords:** brown marmorated stink bug, (*E*)-2-decenal, dispersal, olfaction, transcriptome, odorant-binding protein

## Abstract

*Halyomorpha halys* is a major herbivore insect in the fruit orchards of China that has become a devastating invasive pest in North America and Europe since its accidental introductions in the 1990s and 2000s, respectively. Like other hemipteran insects, *H. halys* releases defensive chemicals against natural enemies, including (*E*)-2-decenal, which is an aldehyde associated with alarm pheromones. In this study, a series of electrophysiological and behavioral tests were conducted to characterize the alarm functions of (*E*)-2-decenal among *H. halys* adults and nymphs. An antennal transcriptome was obtained from a Chinese *H. halys* population, and 44 odorant-binding protein (*OBP*) genes were annotated. Among them, five putative alarm pheromone-binding proteins were screened and were extremely consistent with their homologs from US populations. These five OBPs were later expressed in a heterologous expression system, harvested, and then challenged with (*E*)-2-decenal in a binding assay. All five OBPs showed high binding activities to (*E*)-2-decenal, which demonstrated its behavioral significance as a defensive component in *H. halys*, as well as being the first report of its olfactory reception. These findings will help develop behavioral-mediating tools as part of integrated pest management approaches to control this invasive pest.

## Introduction

Stink bugs (Hemiptera: Pentatomidae) employ a spectrum of volatile compounds that have ecologically significant roles in colonization, courtship, immunity, and chemical defense (Noge et al., 2015; Weber et al., 2017b). Among these compounds, alarm pheromones, which mostly consist of short-chain aliphatic aldehydes, alcohols and esters, alkanes, terpenes, and phenolics, are well-known signature odorant cues in stink bugs (Aldrich, 1988; Millar, 2005; Moraes et al., 2008; Noge and Becerra, 2012). These unpleasant and pungent secretions are stored in the dorsal abdominal glands of nymphs and the metathoracic scent glands of adults and are released under aggressive environmental stimuli to mediate behavioral consequences among individual pentatomids, predators, and natural enemies (Borges and Aldrich, 1992; Weber et al., 2017b). To date, a wide variety of hydrocarbons, aldehydes, and oxo-aldehydes are thought to be defensive compounds involved in the dispersal process (Aldrich, 1988; Tada et al., 2001a,b; Noge and Becerra, 2012; Weber et al., 2017b). Previous studies described the proportions and release biases among species, genders, and developmental stages (Kuhar and Kamminga, 2017; Weber et al., 2017b). A number of key components separated from several stink bug species have multiple functions, including prompting generalist predator avoidance (Noge and Becerra, 2012), delivering antifungal and antibacterial substances (Lopes et al., 2015; Sagun et al., 2016), and accelerating the locating of hosts by parasitoids (Zhong et al., 2017).

*Halyomorpha halys*, the brown marmorated stink bug, is a well-studied stink bug species in the field of chemical ecology (Weber et al., 2017b). Native to Eastern Asia, *H. halys* was accidently introduced into North America and Europe in the 1990s and 2000s, respectively (Haye and Weber, 2017; Valentin et al., 2017; Leskey and Nielsen, 2018). It is now been considered a very damaging invasive insect pest globally (Haye and Weber, 2017). As a highly polyphagous pentatomid herbivore with over 120 host plants, *H. halys* has caused serious annual losses in economically important fruit crops, vegetables, ornamentals, shrubs, and forest trees (Lee et al., 2013). Research has emphasized the identification of its aggregation pheromone, its synergism with the pheromone of *Plautia stali*, and the development of pheromone-based traps for field applications (Borges and Aldrich, 1992; Tada et al., 2001a; Lee et al., 2013; Kuhar and Kamminga, 2017). Additionally, chemical communications among this pest, its host plants, and its natural enemies have been studied to develop biological control based integrated pest management strategies (Bernays and Chapman, 1994; Schoonhoven et al., 2005; Noge and Becerra, 2012; Zhang et al., 2014b; Tognon et al., 2016; Zhong et al., 2017). The defensive chemicals *n*-tridecane, *n*-dodecane, (*E*)-2-decenal, and (*E*)-2-octenal have been identified from *H. halys* volatiles (Kitamura et al., 2008; Noge and Becerra, 2012; Harris et al., 2015; Zhong et al., 2017). In particular, the emission patterns of (*E*)-2-decenal have been investigated in both *H. halys* sexes and 3rd instar nymphs (Harris et al., 2015; Zhong et al., 2017), and it is considered as an alarm pheromone component (Harris et al., 2015; Weber et al., 2017a). However, its alarm effects on *H. halys*, and the molecular basis for the olfactory perception behind its effects, have not been revealed.

*H. halys* senses these semiochemicals through its olfactory organs on the antennae, and odorant-binding proteins (OBPs) play an important role in the olfactory process (Vosshall and Stensmyr, 2005; Leal, 2013; Wicher, 2015; Paula et al., 2016). OBP responses are the first step at the peripheral olfactory level for delivering odorant cues to the transmembrane odorant receptors in the aqueous lumen of chemosensilla on insect antennae (Vogt and Riddiford, 1981). Since the report of the first hemipteran OBP family in *Lygus lineolaris* in 1995, OBPs have been isolated and functionally characterized from over 32 hemipteran species (Dickens et al., 1995; Paula et al., 2016). A recent break-through in *H. halys* reported the annotation of 33 OBPs in its US population, and an olfactory plasticity was observed under alarm pheromone stimulation when males or females were forced to release the alarm pheromone in contained small arena bioassays (Paula et al., 2016). The quantitative assessment of expression changes in *OBP* genes revealed multiple *OBP*s might be involved in detection of alarm pheromone stimulus in *H. halys*. Nevertheless, which genes are responsible for binding alarm pheromone compounds, such as (*E*)-2-decenal, remains unknown. Revealing the existence of (*E*)-2-decenal-sensing OBPs may contribute to new solutions and approaches to control *H. halys* through behavioral interference, such the use of RNA interference technology (Mogilicherla et al., 2018).

It is still critical to understand and demonstrate clear behavioral responses of different developmental stages of *H. halys* to alarm pheromones and their corresponding molecular olfactory mechanisms. (*E*)-2-Decenal is an abundant defensive compound released by *H. halys* adults, and it can strongly repel male and female *H. halys* in Y-tube assays (Zhong et al., 2017). In the present study, we investigated the alarm effects of (*E*)-2-decenal and its olfactory preferences in *H. halys* by conducting electroantennogram (EAG) tests, behavioral assays with grouped or individual *H. halys*, antennal transcriptome analyses, and binding activity assays with putative OBPs. This work provides strong behavioral evidence and molecular olfactory bases to demonstrate the function of (*E*)-2-decenal as an alarm pheromone component in *H. halys*.

## Materials and methods

### Insects

Nymphs and adults of *H. halys* were obtained from laboratory colonies at the MARA-CABI Joint Laboratory, Beijing. The initial laboratory colony was established from wild *H. halys* populations collected in Beijing, China (N40°01′53″; E116°15′32″). They were continuously reared on a diet of organic green beans (*Phaseolus vulgaris* L.) and corn (*Zea mays* L.) in rearing cages (60 × 60 × 60 cm) at 25 ± 1°C, with 65% ± 5% relative humidity and a 16 L:8 D photoperiod (Zhong et al., 2017). Egg masses were collected daily and maintained in separate rearing cages until completion of the nymphal stages. Newly emerged adults were removed and maintained in separate rearing cages as described above. The nymphs and adults of *H. halys* used for the experiments were reared for 2-3 generations in the laboratory.

### Chemicals

n-Hexane (95%, Sigma-Aldrich, St. Louis, MO, USA) was used as the control and solvent, and (*E*)-2-decenal (95%, Sigma-Aldrich, St. Louis, MO, USA) was used as stimulus chemical.

### EAG recordings

EAG recordings were used to identify electrophysiological activities of *H. halys* at each developmental stage (female and male adults, and nymphal larvae). Each antenna of the brown marmorated stink bug was prepared following the standard procedures of cutting its tip and base and immediately mounting the excised antenna between two glass capillary Ag/AgCl electrodes filled with Kaissling saline (Tang et al., 2016). The electrode at the distal end of the antenna was connected through an interface box to a signal acquisition interface board (IDAD; Syntech, Netherlands).

n-Hexane was used as the control, and 1 μg/μl (*E*)-2-decenal was used as the test solution. The measure dose was 10 μg, and the order of antennal exposure was air, n-hexane, and (*E*)-2-decenal. In total, 10 replicates were used. The direct voltage was 2 mv, the continuous flow velocity was 150 ml/min, the stimulated flow velocity was 20 ml/min, the stimulation time was 0.1 s, and the stimulus intervals were 10 s.

The following equation was used to calculate the EAG recording result:

Relative response value = sample response value − control response value.

### Behavioral assays

A group test was performed first to assess the repellency of (*E*)-2-decenal to *H. halys* adults. In total, 10–20 adults were collected and moved into a plastic rearing box for 1 h to let the adults aggregate and rest before the test. A filter paper loaded with 10 μg (*E*)-2-decenal was then put into a syringe, and the volatile was released into the air at a distance of 1 cm from the aggregated *H. halys* adults. The test lasted for 60 s, and the number of dispersed *H. halys* were counted and recorded. n-Hexane was used as the control following the same protocol. In total, 10 replicates were performed for each treatment.

To identify the alarm pheromone activity in each developmental stage of *H. halys* (female and male adults, and 1st to 5th instar nymphs), we performed a behavioral test (Noge et al., 2015) using a gradient of (*E*)-2-decenal solutions (10 μg/μl, 1 μg/μl, 100 ng/μl, 10 ng/μl, and 1 ng/μl) for the nymphs and the last four doses for both genders of adults. A circular track was made by joining the ends of a silicone tube (100 cm long, 1.2 cm inner diameter), and the track was placed on a tripod stand in the behavioral testing laboratory (25°C ± 1°C, relative humidity = 50% ± 10%). An individual *H. halys* was placed gently on the track and left for 5 min until it was motionless. Different 10-μl concentrations of (*E*)-2-decenal were loaded onto a filter paper disk (2 cm diameter), and the disk was then placed 2 mm from the stink bug's antenna. The time taken for the nymph or adult stink bug to change direction and the distance it moved away from the starting point within 15 s were observed and recorded. If the stink bug did not change direction or stayed still for 15 s, the distance and time were recorded as 0 cm and 15 s, respectively. n-Hexane was used as the control. Every developmental stage of *H. halys* for each chemical treatment was tested 15 times.

### Antennal transcriptome analysis and putative OBP annotations

Antennae from *H. halys* were collected, pooled into female and male groups and then used in a standard Illumina HiSeq2000 platform pipeline for *de novo* RNA sequencing at BGI Tech. Co., Beijing, China. Clean reads were obtained using the FastQC tool and then assembled with SOAP. *OBP* annotations were performed using BLAST algorithm-based searches against the NR, NT, Swiss-Prot, KEGG, COG, and GO databases. Translated amino acid sequences of *OBP*s from *H. halys, Apolygus lucorum*, and *Drosophila melanogaster* were first aligned with MUSCLE, and then, a phylogenetic tree was developed using the neighbor-joining method (Saitou and Nei, 1987) in MEGA 7.0.14 software (Kumar et al., 2016). Protein structures of OBPs were predicted with the Swiss-model to locate potential plus-C domains. Alignments of OBP25, OBP30, OBP16, OBP8, and OBP4 between the Chinese and the US populations were performed using the PRALINE multiple sequence alignment online tools.

### Expression and purification of OBP proteins

We chose five *H. halys OPB*s based on the results of a previous study (Paula et al., 2016), which showed that *OBP25, OBP30, OBP16, OPB8*, and *OBP4* were highly expressed after exposure to an alarm pheromone, and the increases in the ranges were ~100,000-, 10,000-, 1,000-, 100-, and 10-fold. We synthesized the genes and heterologously expressed these five *OPB*s. The prokaryotic expression was used to identify the target proteins. In detail, the exact gene sequences from transcriptome analysis were synthesized by the Genscript Biology Company (Nanjing, China) and subcloned into target vector for *Escherichia coli* expression (Table 1).

**Table 1:** Technical details for recombinant OBP protein expressions.

**Protein**	**Vector**	**Expected MW (kDa)**	**Cloning strategy**
OBP25	pET30a	~15.4	NdeI-OBP25-His tag-Stop codon-HindIII
OBP30	pET30a	~13.8	NdeI-OBP30-His tag-Stop codon-HindIII
OPB16	pET30a	~18.5	NdeI-OBP6-His tag-Stop codon-HindIII
OBP8	pET30a	~14.4	NdeI-OBP8-His tag-Stop codon-HindIII
OBP4	pET30a	~12.8	NdeI-OBP4-His tag-Stop codon-HindIII

For each *OBP* gene, recombinant pET30a vectors were transferred into the *E. coli* BL21 (DE3) competent cell strain and allowed to duplicate for 60 min. *E. coli* were then plated on LB agar dishes containing 50 μg/ml kanamycin. Three single cloned colonies were isolated and reared in LB medium until the OD_600_ was 0.6–0.8. Isopropyl β-D-1-thiogalactopyranoside at the final concentration of 1.0 mM was added to two of the three liquid cultures for induction at 15°C for 16 h and at 37°C for 4 h, respectively, with the third sample acting as the negative control. Pilot expression levels of each OBP were detected with SDS-PAGE and western blotting, respectively.

OBP protein samples for later tests were prepared from isopropyl β-D-1-thiogalactopyranoside-induced liquid cultures. The cells were harvested by centrifugation at 12,000 rpm for 20 min at 4°C. Insoluble inclusion cell bodies were dissolved in 8 M urea in 20 mM Tris-HCl buffer at pH 7.4. Raw solutions were then purified through an affinity chromatography XK 16/20 column filled with Ni Sepharose High Performance (GE Healthcare, Little Chalfont, Buckinghamshire, UK). Laddered urea solutions were used for protein renaturation and extensive dialysis. Harvested proteins were then treated with enterokinase (Genscript Biology Company) to cleave His-tag residues. The cleavage proteins were purified following the same protocol as above and then desalted by extensive dialysis, lyophilized, and stored at −70°C until use.

### Competitive fluorescence-binding assay

Emission fluorescence spectra were measured on a HORIBA FluoromMax®-4 fluorescence spectrophotometer in a right-angle configuration with a 1 cm light path quartz cuvette. The protein was dissolved in 50 mM Tris-HCl buffer, pH 7.4, while all ligands used in binding experiments were used as 1 mM methanol solutions. The excitation wavelength was 337 nm, and the range of the scanning emission wavelength was 380–450 nm.

The binding constants of OBPs with probe N-phenyl-1-naphtylamine (1-NPN) were determined. We first added 2 ml 50 mM Tris-HCl solution to the cuvette, then added the OBP until the concentration reached 2 μM, measured and recorded the emission spectrum, continued titration, and recorded the highest stable fluorescence intensity. The Scatchard equation was used to record the binding constant of each OPB with 1-NPN.

The binding abilities of the OBPs and (*E*)-2-decenal were later investigated. Fluorescence intensities were automatically subtracted from blank fluorescence values when harvested from instrument. The excitation wavelength was set at 337 nm, and the range of the scanning emission wavelength was 380–450 nm. First, 2 mL 50-mM Tris-HCl solution was added, and then the OBP and 1-NPN were added until the concentration was 2 μM. The highest stable fluorescence intensity was record. Then, we added the (*E*)-2-decenal, which was dissolved in methanol in the cuvette, and continued the titration from 2 μM until the fluorescence intensity was stable. The highest stable fluorescence intensity was recorded. Each OBP was tested three times. The dissociation constant was calculated based on the formula K_d_ = [IC_50_]/(1 + [1-NPN]/K_1−NPN_), where IC_50_ represents the (*E*)-2-decenal concentration when the fluorescence intensity of [OBP/1-NPN] declined to the half of the highest value, [1-NPN] represents the dissociative concentration of 1-NPN, and K_1−NPN_ represents the dissociation constant of OBP/1-NPN.

### Statistical analysis

All statistics were done in IBM SPSS Statistics 22.0.0 (SPSS, Chicago, IL, USA). Parametric tests (either *t* tests for 2 treatments or GLM for > 2 treatments) were used to compare difference among means for EAG and behavioral assays. Tukey HSD was used for multiple comparison among treatments. For competitive fluorescence-binding assays, correlations and curves were done using Prism 5 for Windows ver. 5.01 (GraphPad software, San Diego, CA, USA). All significance was accepted at the α = 0.05 level. Three replicates were done for binding assays, and means were used for the statistics.

### Data viability

Original EAG voltage data sheets were provided as Data S1. Original competitive fluorescence-binding assay data sheets were provided as Data S2.

## Results

### *(E)-2*-decenal elicited significant electrophysiological responses from *H. halys*

**Figure 1:**
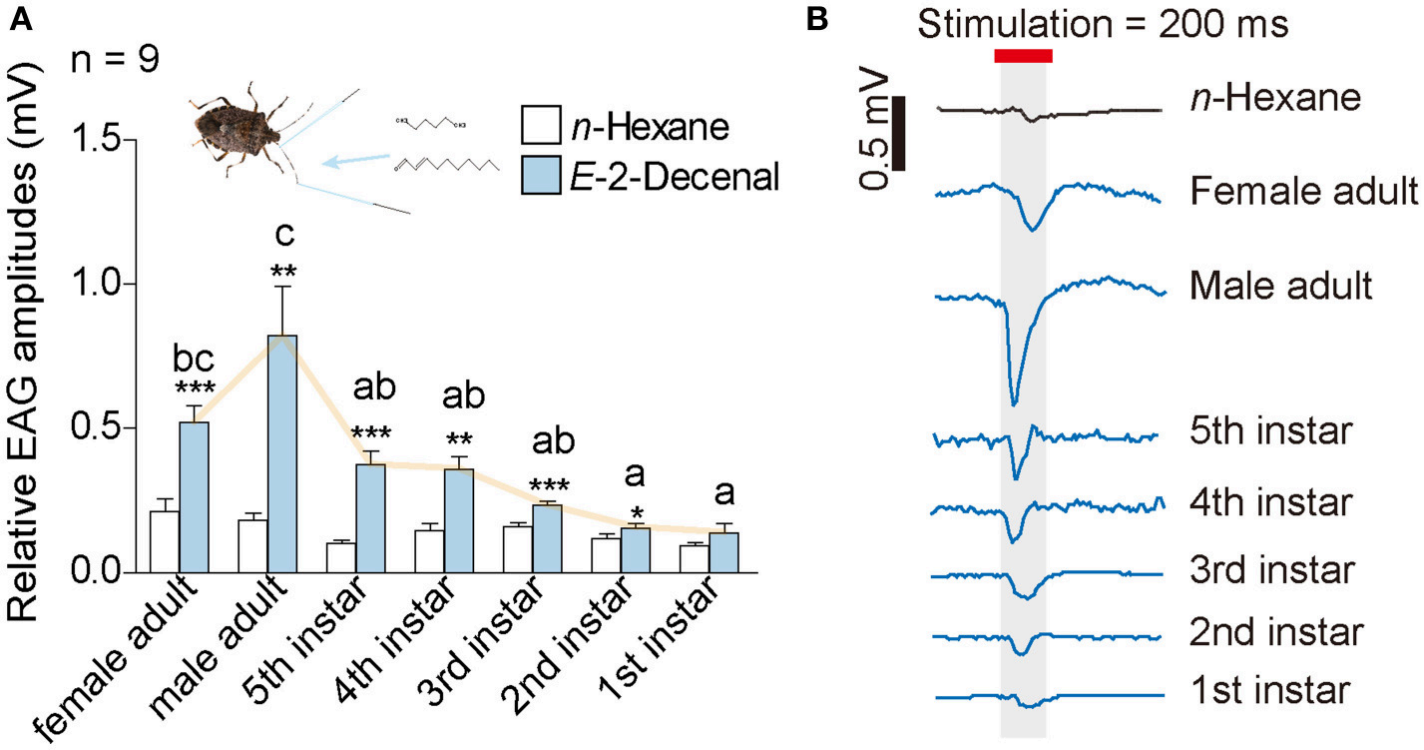
Results from EAG tests of *H. halys*. **(A)** A dosage of 10 μg (*E*)-2-decenal was used as treatment, and 10 μl n-hexan was used as control. Lower case letters indicate significant differences among *H. halys* stages in terms of EAG responses toward (*E*)-2-decenal [GLM and Tukey HSD, *F*_(6, 61)_ = 10.09, *P* < 0.0001]. Asterisks indicate significant higher EAG responses of *H. halys* antennae toward (*E*)-2-decenal compared with n-hexane (*t*-tests, female adult: *t* = 7.7, *P* < 0.0001; male adult: *t* = 3.9, *P* = 0.0036; fifth instar nymph: *t* = 6.0, *P* = 0.0002; fourth instar nymph: *t* = 3.4, *P* = 0.0086; third instar nymph: *t* = 5.3, *P* = 0.0005; second instar nymph: *t* = 2.6, *P* = 0.03; first instar nymph: *t* = 1.9, *P* = 0.091). **(B)** Example traces of EAG responses from each stage and gender of *H. halys*. Original voltage data can be found in Data S1.

We first conducted EAG tests to investigate the biological activities of (*E*)-2-decenal at the antennal level. Among all of the *H. halys*' developmental stages, we observed significantly greater electrophysiological responses compared with the n-hexane control, except for the 1st instar nymphs (Figure 1). Antennae of adults had greater EAG responses than nymphs. Moreover, (*E*)-2-decenal stimulated significantly greater EAG responses in male adults compared with all nymphs, but there was no significant difference between the sexes of adults. Female *H. halys* showed significantly greater responses compared with 1st and 2nd instar nymphs (Figure 1, Data S1).

### *(E)-*2-decenal dispersed all of the developmental stages of *H. halys* in a temporospatial manner

**Figure 2:**
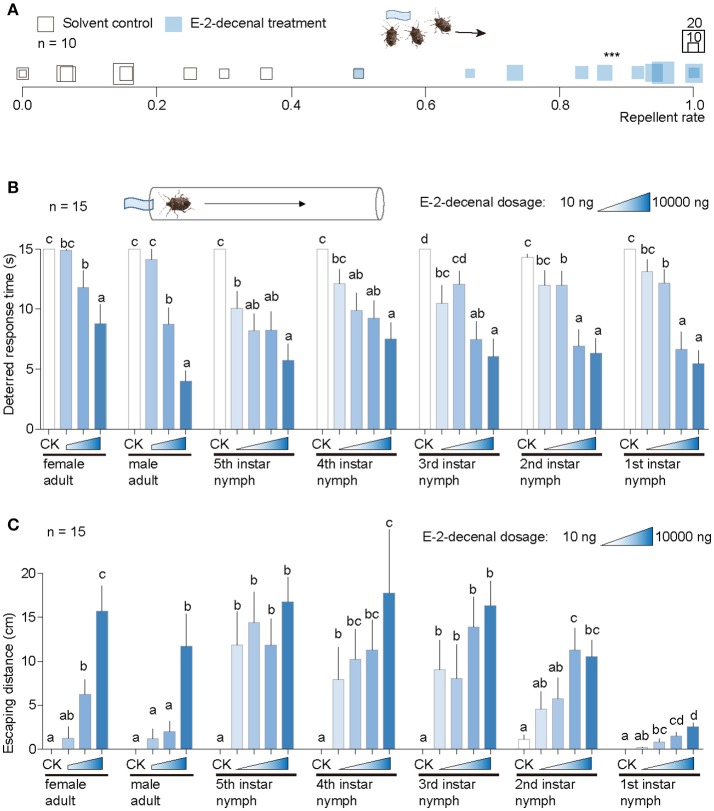
Behavioral responses of *H. halys* to (*E*)-2-decenal. **(A)** Results from a grouped test with 30 *H. halys* for each replicate. (*E*)-2-decenal was used at a dosage of 10 μg and solvent n-hexane was used as control. Square sizes indicate aggregated numbers of *H. halys* adults. Repellent rates for (*E*)-2-decenal and control were 84.1 ± 5.14% and 18.47 ± 5.25%, respectively. ^***^Indicates significant difference was observed between (*E*)-2-decenal and control [student's *t*-test, *t*_(18)_ = 2.1, *P* < 0.001]. **(B)** Comparison of deterred response times of single *H. halys* in each developmental stage among (*E*)-2-decenal dosages. Solvent was used as control (CK). Different lower case letters indicate significant differences were observed among dosages in each developmental stage of *H. halys* [GLM and Tukey HSD, female adults: *F*_(3, 86)_ = 15.0, *P* < 0.0001; male adults: *F*_(3, 86)_ = 63.03, *P* < 0.0001; fifth instar nymphs: *F*_(4, 115)_ = 26.2, *P* < 0.0001; fourth instar nymphs: *F*_(4, 115)_ = 18.17, *P* < 0.0001; third instar nymphs: *F*_(4, 115)_ = 24.88, *P* < 0.0001; second instar nymphs: *F*_(4, 115)_ = 20.98, *P* < 0.0001; first instar nymphs: *F*_(4, 115)_ = 40.11, *P* < 0.0001]. Error bars indicate + s.e.m. **(C)** Comparison of escaping distances of single *H. halys* in each developmental stage among (*E*)-2-decenal dosages. Solvent was used as control (CK). Different lower case letters indicate significant differences were observed among dosages in each developmental stage of *H. halys* [GLM and Tukey HSD, female adults: *F*_(3, 86)_ = 32.15, *P* < 0.0001; male adults: *F*_(3, 86)_ = 13.36, *P* < 0.0001; fifth instar nymphs: *F*_(4, 115)_ = 18.5, *P* < 0.0001; fourth instar nymphs: *F*_(4, 115)_ = 13.62, *P* < 0.0001; third instar nymphs: *F*_(4, 115)_ = 14.55, *P* < 0.0001; second instar nymphs: *F*_(4, 112)_ = 11.05, *P* < 0.0001; first instar nymphs: *F*_(4, 115)_ = 22.78, *P* < 0.0001]. Error bars indicate + s.e.m.

In a grouped dispersal tests with 30 *H. halys* adults, (*E*)-2-decenal caused significantly greater dispersal rates among tested replicates (Movie S1) than the n-hexane control, while the group treated with the latter showed little behavioral responses within 1 min (Movie S2). (*E*)-2-Decenal significantly influenced the behaviors of *H. halys* adults compared with n-hexane control (Figure 2A).

We then used a route test to assess in detail the behavioral responses of *H. halys* individuals toward (*E*)-2-decenal. *H. halys* at each developmental stage were sensitive to this chemical, with the repellent effect starting at a tested dose of 10 ng to 5th instar nymphs (Figure 2B). All stages of *H. halys* were significantly repelled by 1 μg and 10 μg (*E*)-2-decenal compared with the n-hexane control. The exposure time prior to the initiation of escape behaviors significantly decreased as the dose of (*E*)-2-decenal increased in all tested stink bug stages (Figure 2B).

*Halyomorpha halys* nymphs moved farther to escape the chemical source compared with adults, excepted for the 1st instar nymphs. In particular, significantly longer moving distances were observed in 3rd to 5th instar nymphs for all tested doses of (*E*)-2-decenal compared with the n-hexane control (Figure 2C). Moving distances generally increased as the tested (*E*)-2-decenal concentration increased (Figure 2C). In comparison with other developmental stages, the 1st instar nymphs moved shorter distances at all tested doses (Figure 2C).

### Five OBPs were screened as putative alarm pheromone-binding proteins

**Figure 3:**
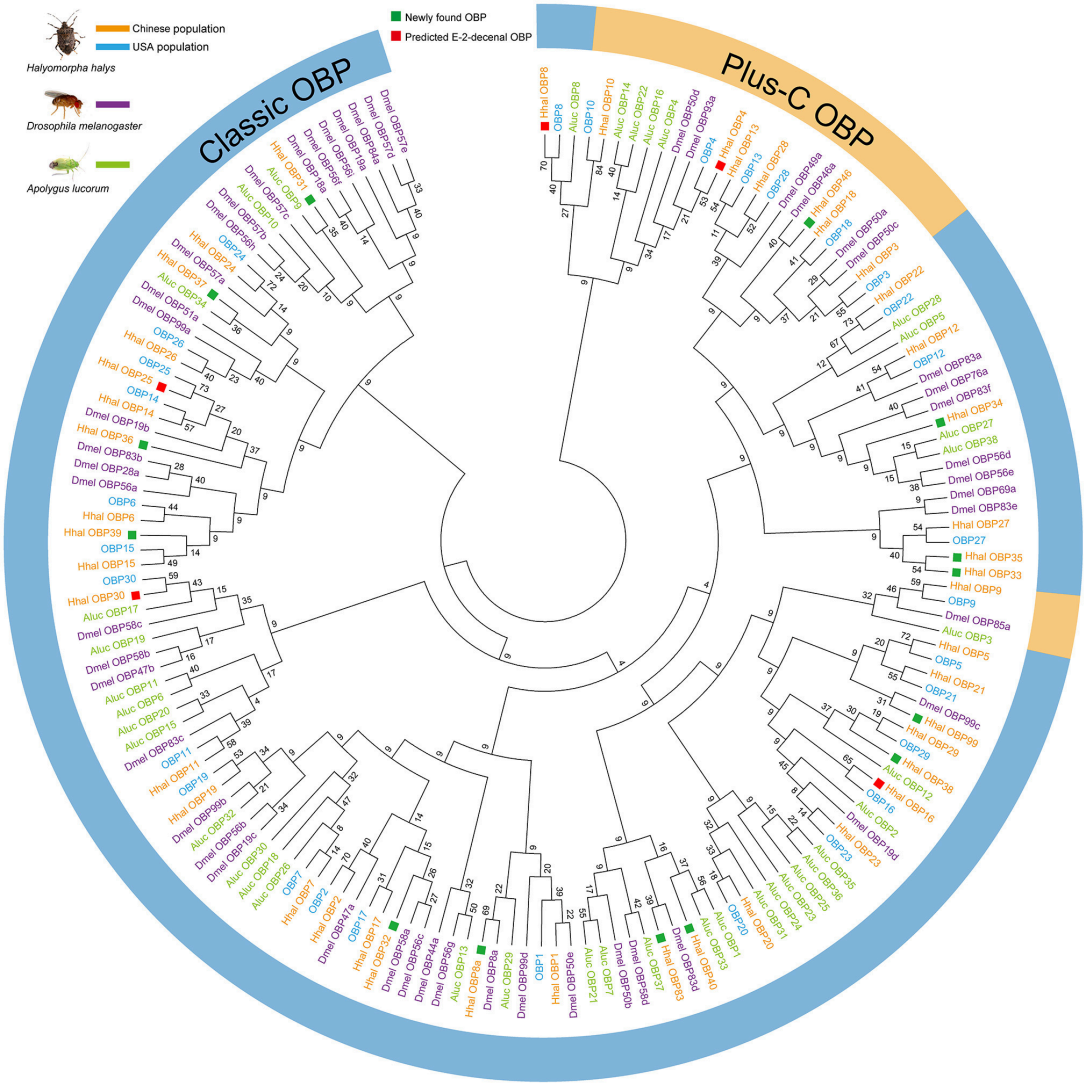
Neighbor-Joining phylogenetic tree established from candidate *OBP* sequences from two *H. halys* populations and other two insect species. The evolutionary history was inferred using the Neighbor-Joining method. The optimal tree with the sum of branch length = 95.69757843 is shown. The evolutionary distances were computed using the number of differences method and are in the units of the number of amino acid differences per sequence. The analysis involved 163 amino acid sequences. All positions containing gaps and missing data were eliminated. There were a total of 4 positions in the final dataset. Evolutionary analyses were conducted in MEGA7. Green squares showed 14 newly annotated *OBPs* in antennal transcriptome from Chinese strain of *H. halys*. Red squares indicate putative (*E*)-2-decenal binding *OBPs* (*HhalOBP25, HhalOBP30, HhalOBP16, HhalOBP8*, and *HhalOBP4*). Included are *OBP* genes identified from: *Halyomorpha halys* (Hhal: Chinese strain; OBP: USA strain), *Apolygus lucorum* (Aluc), and *Drosophila melanogaster* (Dmel). Outside circle shows predicted classic *OBPs* and plus-C *OBPs*.

In total, 44 *OBP*s were annotated in the *H. halys* antennal transcriptome. A phylogenic tree was established using OBPs from both Chinese and US populations of *H. halys* (Paula et al., 2016), *D. melanogaster* (Hekmat-Scafe et al., 2002), and *A. lucorum* (Yuan et al., 2015). In total, 14 *OBP*s, *OBP8a, OBP31, OBP32, OBP33, OBP34, OBP35, OBP36, OBP37, OBP38, OBP39, OBP40, OBP46, OBP83*, and *OBP99*, were newly identified in the Chinese *H. halys* populations (Figure 3). By aligning the amino acid sequences of *H. halys* OBPs, we found that 36 out of 44 OBPs contained the normal six conserved Cys residues, indicating that the predicted C-pattern motif was indicative of a classic OBP in insects (Xu et al., 2009). OBP46 and seven other previously reported OBPs (Paula et al., 2016) had additional conserved Cys residues and were classified as Plus-C OBPs (Figure 3).

**Figure 4:**
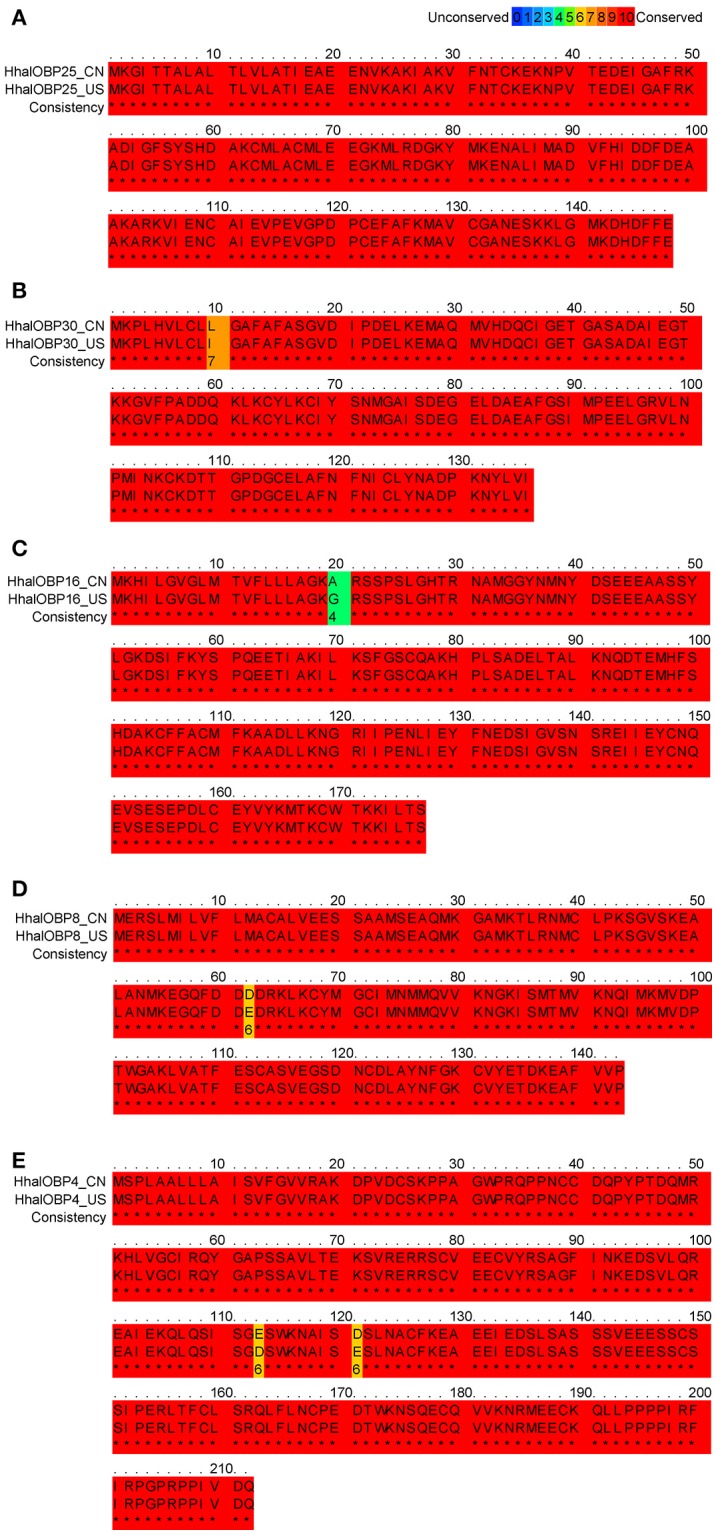
Multiple alignments of *H. halys OBP25, OBP30, OBP16, OBP8*, and *OBP4*. **(A)** Alignment of *H. halys OBP25* amino acid sequences between the Chinese (CN) and the American (US) populations. The amino acid identity of the two populations was 100%. **(B)** Alignment of *H. halys OBP30* amino acid sequences between the Chinese (CN) and the American (US) population. The amino acid identity of the two populations was 99%. **(C)** Alignment of *H. halys OBP16* amino acid sequences between the Chinese (CN) and the American (US) populations. The amino acid identity of the two populations was 99%. **(D)** Alignment of *H. halys OBP8* amino acid sequences between the Chinese (CN) and the American (US) populations. The amino acid identity of the two populations was 99%. **(E)** Alignment of *H. halys OBP4* amino acid sequences between the Chinese (CN) and the American (US) populations. The amino acid identity of the two populations was 99%. Letters in red boxes (^*^) represent fully conserved amino acids while unconserved residues were shown by different colors.

The expression levels of *OBP25, OBP30, OBP16, OBP8*, and *OBP4* in US *H. halys* populations greatly increase after exposure to an alarm pheromone (Paula et al., 2016). We successfully identified homologs for all five of these *OBP*s in Chinese populations (Figure 3). Moreover, all five *OBP*s had conserved amino acid sequences between US and Chinese populations, with identity levels > 99% (Figure 4). The highly conserved amino acid sequences of these five *OBP*s indicated that they may also be functionally conserved among different populations. Both behavioral assays and chemical analyses revealed that (*E*)-2-decenal is the main functional alarm ingredient (Zhong et al., 2017), which suggests that *OBP25, OBP30, OBP16, OBP8*, and *OBP4* in *H. halys* may contribute to the olfactory reception of (*E*)-2-decenal. We selected these five *OBP*s to further study their functional characteristics and binding activities to this chemical.

### Expression and purification of OBP25, OBP30, OBP16, OBP8, and OBP4

**Figure 5:**
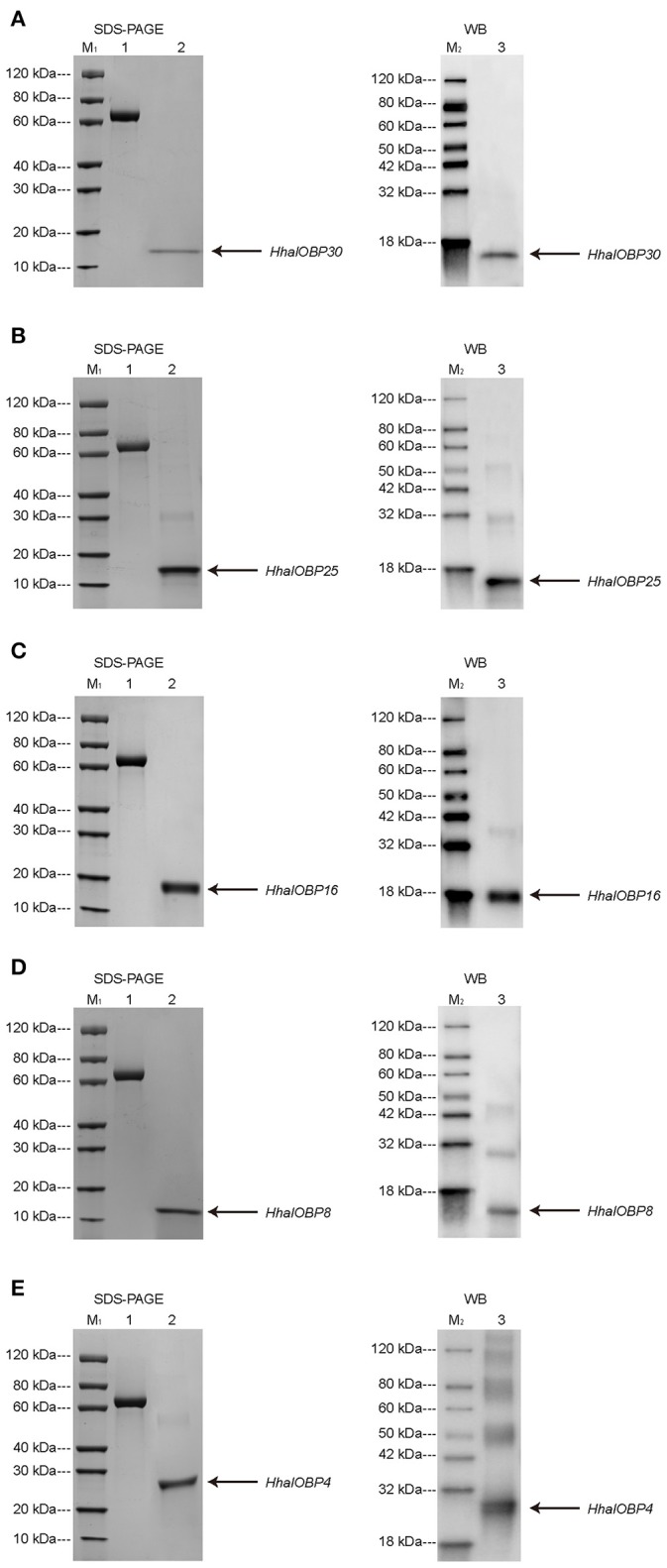
SDS-PAGE (left) and Western blot (right) analysis for purified *H. halys OBP30* **(A)**, *H. halys OBP25* **(B)**, *H. halys OBP16* **(C)**, *H. halys OBP8* **(D)**, and *H. halys OBP4* **(E)** expression. Arrows indicate the protein bands. M_1_ and M_2_ were molecular markers. 1: bovine serum protein as a reference. 2 & 3: purified OBPs.

The chosen five *OBP* genes were duplicated *in vitro* through a bacterial expression system. The purification was performed following a standard protocol (Prestwich, 1993; Calvello et al., 2003). After the cleavage of the His-tag and purification, sodium dodecyl sulfate-polyacrylamide gel electrophoresis (SDS-PAGE) and western blotting showed that the molecular masses of *OBP25, OBP30*, and *OBP8* were ~15 kDa, while those of *OBP16* and *OBP4* were ~18 and 28 kDa, respectively. The molecular mass information obtained from SDS-PAGE and western blot tests were consistent with the amino acid lengths predicted from the *H. halys* antennal transcriptome (Figure 5).

### Binding activities of OBP25, OBP30, OBP16, OBP8, and OBP4 to (*E*)-2-decenal

**Figure 6:**
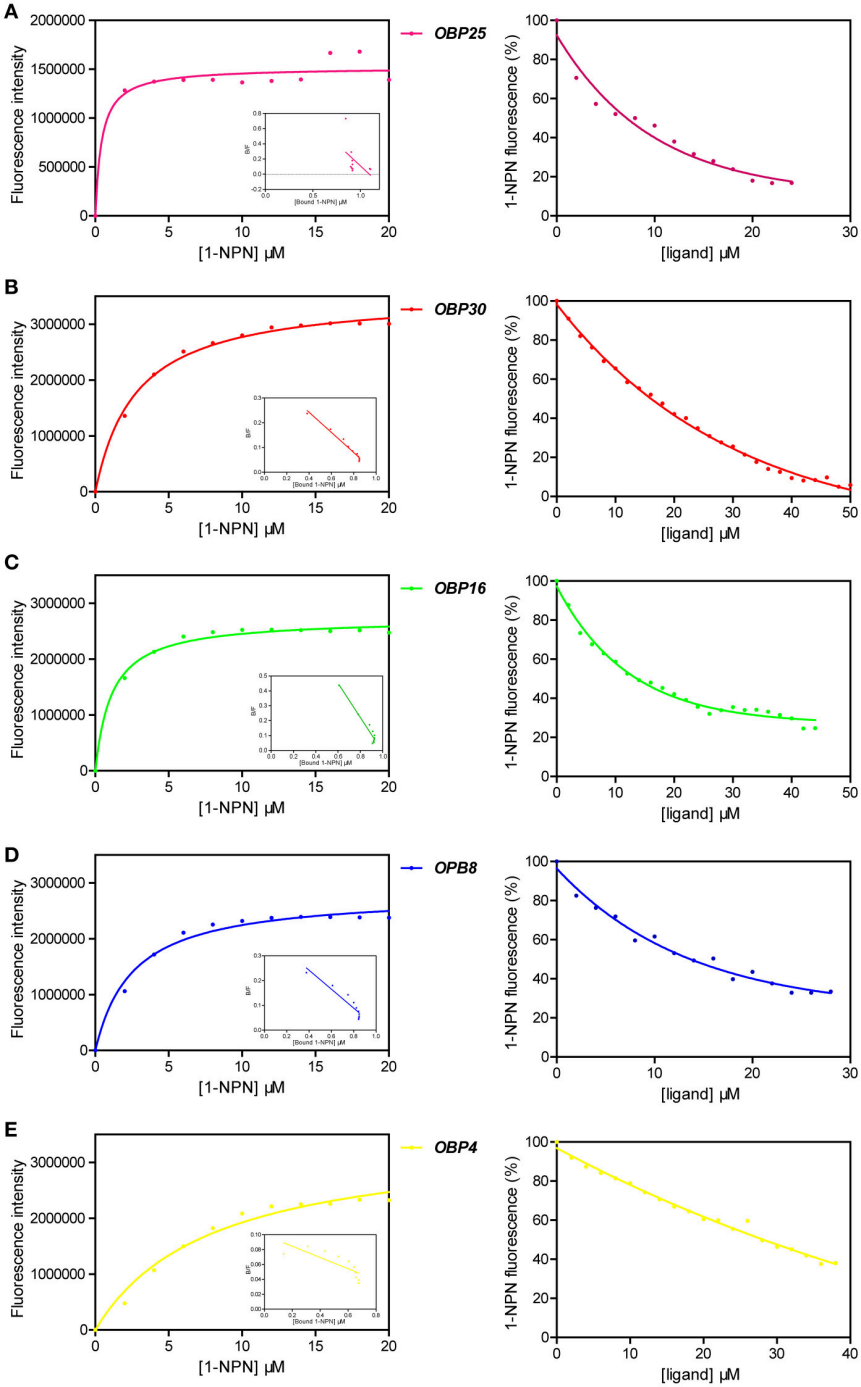
Competitive fluorescence binding assays of *H. halys OBPs* for (*E*)-2-decenal ligand, with 1-NPN as the fluorescent probe. Detailed calculation for key measurements were listed in Table 2. Means from 3 replicates were used in the plotting and original data sheets were provided in Data S2. **(A)** Left: binding curve of 1-NPN to *H. halys OBP25* and the resulting Scatchard plot. Right: competitive binding curve of (*E*)-2-decenal to *OBP25*. **(B)** Left: binding curve of 1-NPN to *H. halys OBP30* and the resulting Scatchard plot. Right: competitive binding curve of (*E*)-2-decenal to *OBP30*. **(C)** Left: binding curve of 1-NPN to *H. halys OBP16* and the resulting Scatchard plot. Right: competitive binding curve of (*E*)-2-decenal to *OBP16*. **(D)** Left: binding curve of 1-NPN to *H. halys OBP8* and the resulting Scatchard plot. Right: competitive binding curve of (*E*)-2-decenal to *OBP8*. **(E)** Left: binding curve of 1-NPN to *H. halys OBP4* and the resulting Scatchard plot. Right: competitive binding curve of (*E*)-2-decenal to *OBP4*.

The fluorescent probe 1-NPN was first used to bind the *OBP*s. Significant increases in fluorescence intensity levels were observed when the *OBP*s were added. Among the tested *OBP*s, *OBP25* revealed the highest slope, yet was saturated at the lowest intensity. The 1-NPN dissociation constants for the *OBP*s were calculated from correlation curves as shown in Table 2 (Figures 6A–E, left column, Data S2).

**Table 2:** Result summary of competitive fluorescence-binding assays.

**Protein**	**K_OBPx/1-NPN_ (μM)**	**K_d_ (μM)**	**IC_50_ (μM)**
OBP25	0.51 ± 0.30	2.21 ± 0.064	7.18 ± 0.21
OBP30	2.79 ± 0.74	10.53 ± 0.64	16.69 ± 1.01
OPB16	1.13 ± 0.08	6.16 ± 0.42	13.72 ± 0.94
OBP8	2.74 ± 0.80	8.61 ± 0.24	13.71 ± 0.38
OBP4	9.18 ± 2.67	26.04 ± 3.01	31.33 ± 3.61

We then challenged each OBP/1-NPN complex with (*E*)-2- decenal to test their binding affinities to this alarm pheromone component. All five OBPs bound to (*E*)-2-decenal (Table 2, Figure 6). The top four highly expressed OBPs increased from 100,000- to 100-fold (Paula et al., 2016) when *H. halys* was treated with the alarm pheromone mixture, and they all showed excellent binding activities to (*E*)-2-decenal, with IC50 values < 32 μM and Kd values < 20 μM. However, OBP4 increased only by 10-fold and did not show a good binding activity to (*E*)-2-decenal. Thus, (*E*)-2-decenal is likely a major ligand of OBP25, OBP30, OBP16, and/or OBP8, but OBP4 may be a general-binding protein that uses (*E*)-2-decenal as only a minor ligand. The functions of these five OBPs may be essential for *H. halys* to successfully initially detect (*E*)-2-decenal as an alarm signal.

## Discussion

In the current study, behavioral responses of *H. halys* elicited by (*E*)-2-decenal as an alarm pheromone were revealed through comprehensive electrophysiological tests and laboratory bioassays. However, although greater EAG responses were observed in adult *H. halys*, they did not show a corresponding greater behavioral sensitivity to low doses in comparison with nymphs. This may result from differences in peripheral or central coding patterns among the developmental stages of the insect (Hansson and Stensmyr, 2011; Junker et al., 2017; Haverkamp et al., 2018). (*E*)-2-Decenal may be sensed through combinatorial coding that decides its final behavioral effects on adult *H. halys*, which suggests that it may have other functional roles in adults (Haverkamp et al., 2018). Because EAG tests can only record the overall electrophysiological responses from the whole antenna, further investigations on (*E*)-2-decenal sensilla's sensing diversity and distributional mapping will be needed. Moreover, compared with adults, *H. halys* nymphs were more sensitive to (*E*)-2-decenal as assessed by reaction times and escape distances from the chemical source. The exceptional was 1st instar nymphs, which might be attributed to their incomplete neurological systems development (Fu et al., 2013). According to previously reported works, 3rd instar nymphs emit a greater level of (*E*)-2-decenal than adult males or females (Harris et al., 2015). Studies on other pentatomids indicate that nymphal defensive secretions are enriched in high-molecular weight constituents, presumably because the flightless immature stages require longer-lasting protection (Aldrich, 1988). Thus, we hypothesized that the greater sensitivity of nymphal *H. halys*, except 1st instars, to (*E*)-2-decenal helps them disperse quickly and avoid threats from natural enemies in the environment, resulting in higher survival rates.

(*E*)-2-Decenal has also been identified as a component of defensive secretions in other pentatomids, such as *Nezara viridula* (Lockwood and Story, 1987), *Erthesina fullo* (Kou et al., 1989), *Chinavia impicticornis*, and *Chinavia ubica* (Pareja et al., 2007). However, (*E*)-2-decenal had no alarm effects on adult *N. viridula* as assessed by the olfactometer assay (Lockwood and Story, 1987), and to our knowledge, its exact alarm function has not been clarified in other pentatomids, except our present study. Exposure to (*E*)-2-decenal acted as a strong feeding deterrent for *H. halys* in laboratory trials when it was combined with tridecane (Zhang et al., 2014a), which is a major component of the defensive chemicals emitted by *H. halys* (Harris et al., 2015; Zhong et al., 2017). Moreover, (*E*)-2-decenal has a fungistatic effect and inhibits spore germination in entomopathogenic fungi (Weber et al., 2017b).

In the antennal transcriptome analysis, we found 14 more *OBP* genes in the Chinese *H. halys* populations. As the pest originated from East Asia, this result is not surprising because geographically ancient populations usually maintain a higher divergence in the relevant functional protein abundance owing to the longer evolutionary process and a broader genomic variation in populations (Gariepy et al., 2014; Zhu et al., 2016). Genetic analysis also showed much lower genetic diversity in US *H. halys* populations compared to Chinese populations (Xu et al., 2013). Interestingly, the screened amino acid sequences of OBP25, OBP30, OBP16, OBP8, and OBP4 were highly conserved between a Chinese and US population but not completely the same. However, because (*E*)-2-decenal exists in both populations (Harris et al., 2015; Zhong et al., 2017), we expect that these OBPs will be functionally conserved in both populations.

According to the slopes of the competitive binding assays, the order of the tested OBPs binding activities to (*E*)-2-decenal was OBP25 > OBP16 > OBP 30 > OBP 8 > OBP4. As OBP25 was observed in the binding assays to have the highest affinity for (*E*)-2-decenal, this protein may be involved in a rapid response pathway that drives the initial escape behavior of *H. halys* within a short time period after exposure to low levels of (*E*)-2-decenal. The other four OBPs, which have weaker affinities for (*E*)-2-decenal, are therefore less sensitive, and they could be involved in detecting higher levels of (*E*)-2-decenal.

In addition, OBPs usually do not act exclusively as odorant receptors and may have a spectrum of chemical ligands (Leal, 2013; Larter et al., 2016). The OBPs with lower increases in expression, such as OBP16, OBP8, and OBP4, may also serve as more general carriers that deal with multiple semiochemicals, such as other components in the alarm pheromone blends. Moreover, the newly annotated 14 OBPs may contribute to (*E*)-2-decenal olfactory sensing; therefore, it is desirable to further investigate their olfactory functions.

In summary, our results have revealed the clear behavioral effects of (*E*)-2-decenal on adult *H. halys* of both sexes and the nymphal stages, and this alarm pheromone component was sensed by olfactory reception through OBP25, OBP30, OBP16, OBP8, and OBP4. (*E*)-2-Decenal mediates the host-finding and oviposition behaviors of *Trissolcus japonicas*, an egg parasitoid of *H. halys* (Zhong et al., 2017). Therefore, the information regarding these five *H. halys* OBPs provides insights that can be used to identify potential (*E*)-2-decenal OBPs in *T. japonicas* wasps. Because of the higher tolerance of adult *H. halys* to (*E*)-2-decenal and the discovery of diversified candidate OBPs, we hypothesize that the chemosensitization of *H. halys* to (*E*)-2-decenal through its olfactory system is complex. The current study contributes to the understanding of *H. halys*' olfactory perception of its alarm pheromone components, but further studies are needed to determine the complete olfactory mechanism. Such knowledge of an alarm pheromone will be potentially useful in developing behavioral-mediating tools as part of integrated pest management programs to control this pest in native and invaded regions.

## Author contributions

FZ, G-HC, J-PZ, and RT designed the study. Y-ZZ and J-PZ conducted the behavioral assays. Y-ZZ and RT conducted the electrophysiological and molecular tests. K-LH and Z-YW helped in carrying out the molecular work. S-YY and RT conducted the antennal transcriptome analysis. Y-ZZ, RT, and FZ analyzed the data and drafted the manuscript with inputs provided from all.

### Conflict of interest statement

The authors declare that the research was conducted in the absence of any commercial or financial relationships that could be construed as a potential conflict of interest.
