# Supplementary material for: Behavioral Evidence and Olfactory Reception of a Single Alarm Pheromone Component in Halyomorpha halys
Source: Front Physiol. 2018 Nov 13;9:1610. doi: 10.3389/fphys.2018.01610 (PMC6243750; doi:10.3389/fphys.2018.01610)
Supplement: Supplementary file 3 [file Data_Sheet_1.PDF]

EAG responses to n-hexane (mV)

Adult		Nymph				
Female	Male	5th	4th	3rd	2nd	1st
0.402	0.28	0.16	0.286	0.175	0.17	0.169
0.456	0.312	0.1	0.18	0.169	0.14	0.079
0.185	0.214	0.148	0.115	0.243	0.18	0.117
0.162	0.167	0.09	0.22	0.172	0.068	0.129
0.356	0.23	0.08	0.1	0.119	0.085	0.086
0.116	0.128	0.081	0.12	0.14	0.095	0.068
0.098	0.15	0.1	0.1	0.203	0.153	0.06
0.182	0.146	0.05	0.065	0.11	0.039	0.062
0.03	0.098	0.08	0.14	0.167	0.1	0.065
0.135	0.11	0.131		0.104	0.158	

EAG responses to E2D (mV)

Adult		Nymph				
Female	Male	5th	4th	3rd	2nd	1st
0.637	0.998	0.606	0.317	0.32	0.211	0.323
0.77	1.646	0.21		0.238	0.169	0.246
0.694	0.817	0.213	0.205	0.256	0.201	0.162
0.516	0.535	0.463	0.293	0.245	0.079	0.162
0.705	0.441	0.536	0.238	0.25	0.095	0.103
0.302	1.663	0.421	0.365	0.237	0.128	0.109
0.603	1.243	0.41	0.394	0.213	0.132	0.066
0.326	0.335	0.227	0.498	0.162	0.124	0.039
0.348	0.246	0.2	0.621	0.229	0.239	0.032
0.319	0.294	0.463	0.298	0.182	0.176	
