# Supplementary material for: Behavioral Evidence and Olfactory Reception of a Single Alarm Pheromone Component in Halyomorpha halys
Source: Front Physiol. 2018 Nov 13;9:1610. doi: 10.3389/fphys.2018.01610 (PMC6243750; doi:10.3389/fphys.2018.01610)
Supplement: Supplementary file 4 [file Data_Sheet_2.PDF]

OBP25 binding with 1-NPN

1-NPN con.	Rep 1	Rep 2	Rep 3	Mean
0	0	0	0	0
2	1214630	1367540	1272690	1284953
4	1306730	1536040	1279590	1374120
6	1328030	1529440	1313190	1390220
8	1328630	1533740	1317490	1393287
10	1296130	1513540	1287390	1365687
12	1329630	1515140	1298990	1381253
14	1328730	1546440	1312290	1395820
16	1334730	1535140	2132090	1667320
18	1325130	1537850	2181090	1681357
20	1327820	1539270	1308490	1391860

OBP30 binding with 1-NPN

1-NPN con.	Rep 1	Rep 2	Rep 3	Mean
0	0	0	0	0
2	1406230	1383530	1282400	1357387
4	2282750	2052870	1960960	2098860
6	2878260	2401270	2250160	2509897
8	3201830	2427670	2351960	2660487
10	3358330	2606970	2427460	2797587
12	3669630	2693770	2461160	2941520
14	3718930	2723970	2484460	2975787
16	3827630	2725470	2485660	3012920
18	3816830	2737770	2479060	3011220
20	3813030	2728370	2475860	3005753

OBP16 binding with 1-NPN

1-NPN con.	Rep 1	Rep 2	Rep 3	Mean
0	0	0	0	0
2	2195310	1472750	1314410	1660823
4	2799010	1859950	1734410	2131123
6	3153510	2097950	1965610	2405690
8	3235310	2175250	2039410	2483323
10	3289610	2187150	2096110	2524290
12	3290110	2197350	2096910	2528123
14	3277210	2181350	2093610	2517390
16	3257710	2165550	2083210	2502157
18	3255760	2175690	2112430	2514627
20	3204590	2168680	2053750	2475673

OBP8 binding with 1-NPN

1-NPN con.	Rep 1	Rep 2	Rep 3	Mean
0	0	0	0	0
2	425500	1473890	1280240	1059877
4	1407330	2112090	1627740	1715720
6	1860030	2412990	2050540	2107853
8	2048130	2520090	2191840	2253353
10	2132830	2565490	2254740	2317687
12	2159230	2597090	2364040	2373453
14	2168830	2608090	2395440	2390787
16	2164930	2606790	2393040	2388253
18	2154530	2602290	2391440	2382753
20	2144580	2594850	2390420	2376617

OBP4 binding with 1-NPN

1-NPN con.	Rep 1	Rep 2	Rep 3	Mean
0	0	0	0	0
2	677540	655460	94880	475960
4	1261640	1234020	711650	1069103
6	1687020	1712450	1097320	1498930
8	2057440	2067170	1338920	1821177
10	2321440	2356780	1570720	2082980
12	2297640	2601580	1736920	2212047
14	2250840	2734780	1763620	2249747
16	2176940	2762180	1841120	2260080
18	2169850	2928580	1895420	2331283
20	2168950	2876880	1927020	2324283

OBP25 binding with E2D

E2D con.	Rep 1	Rep 2	Rep 3	Mean	%	
0	1418670	1444520	1543900	1469030	1469030	100.00%
2	1022940	1067710	1022380	1037677	1469030	70.64%
4	869420	867900	786900	841406.7	1469030	57.28%
6	797580	724700	773940	765406.7	1469030	52.10%
8	744400	706730	752890	734673.3	1469030	50.01%
10	633540	698370	702830	678246.7	1469030	46.17%
12	370670	659300	647340	559103.3	1469030	38.06%
14	365840	458130	568350	464106.7	1469030	31.59%
16	333100	404830	496460	411463.3	1469030	28.01%
18	325480	373840	353820	351046.7	1469030	23.90%
20	246140	263940	285350	265143.3	1469030	18.05%
22	246530	255490	237490	246503.3	1469030	16.78%
24	247100	256830	240620	248183.3	1469030	16.89%

OBP30 binding with E2D

E2D con.	Rep 1	Rep 2	Rep 3	Mean		%
0	1255780	1381460	1676910	1438050	1438050	100.00%
2	1163870	1254410	1505360	1307880	1438050	90.95%
4	1014320	1168880	1355390	1179530	1438050	82.02%
6	938700	1102130	1246520	1095783	1438050	76.20%
8	852920	948340	1182990	994750	1438050	69.17%
10	792880	926730	1104850	941486.7	1438050	65.47%
12	751060	850990	918630	840226.7	1438050	58.43%
14	720440	806600	857490	794843.3	1438050	55.27%
16	686760	727860	830530	748383.3	1438050	52.04%
18	610520	746160	692330	683003.3	1438050	47.50%
20	543460	655520	617840	605606.7	1438050	42.11%
22	557540	592630	578690	576286.7	1438050	40.07%
24	435520	553910	514090	501173.3	1438050	34.85%
26	374780	539440	417440	443886.7	1438050	30.87%
28	352040	453300	384900	396746.7	1438050	27.59%
30	290140	441650	369040	366943.3	1438050	25.52%
32	256480	397960	269300	307913.3	1438050	21.41%
34	245000	315660	201050	253903.3	1438050	17.66%
36	176020	296850	134670	202513.3	1438050	14.08%
38	153900	275850	110060	179936.7	1438050	12.51%
40	91680	258030	53850	134520	1438050	9.35%
42	80340	204810	64930	116693.3	1438050	8.11%
44	82530	159040		120785	1438050	8.40%
46		140040		140040	1438050	9.74%
48		71000		71000	1438050	4.94%
52		83640		83640	1438050	5.82%

OBP16 binding with E2D

E2D con.	Rep 1	Rep 2	Rep 3	Mean		%
0	2616240	3333100	3072520	3007287	3007287	100.00%
2	2314180	3029730	2571860	2638590	3007287	87.74%
4	2046700	2332750	2241150	2206867	3007287	73.38%
6	1803680	2079580	2219920	2034393	3007287	67.65%
8	1667490	2025240	1989530	1894087	3007287	62.98%
10	1587330	1877100	1830370	1764933	3007287	58.69%
12	1380750	1751630	1614220	1582200	3007287	52.61%
14	1133060	1707050	1607240	1482450	3007287	49.30%
16	1131050	1640030	1560020	1443700	3007287	48.01%
18	1015240	1570730	1501720	1362563	3007287	45.31%
20	928690	1467360	1407310	1267787	3007287	42.16%
22	859710	1329660	1338450	1175940	3007287	39.10%
24	771590	1211850	1228120	1070520	3007287	35.60%
26	513200	1197600	1176490	962430	3007287	32.00%
28	717240	1202890	1137600	1019243	3007287	33.89%
30			1067060	1067060	3007287	35.48%
32			1021840	1021840	3007287	33.98%
34			1027150	1027150	3007287	34.16%
36			996300	996300	3007287	33.13%
38			946430	946430	3007287	31.47%
40			892400	892400	3007287	29.67%
42			737390	737390	3007287	24.52%
44			741770	741770	3007287	24.67%

OBP8 binding with E2D

E2D con.	Rep 1	Rep 2	Rep 3	Mean		%
0	2.21E+06	1875410	2000870	2.03E+06	2027150	100.00%
2	1902250	1604970	1510110	1.67E+06	2027150	82.50%
4	1726980	1422790	1492400	1.55E+06	2027150	76.33%
6	1651130	1381030	1336490	1.46E+06	2027150	71.84%
8	1458570	1040340	1127340	1.21E+06	2027150	59.63%
10	1356850	1114650	1277080	1.25E+06	2027150	61.64%
12	1239570	871200	1118280	1.08E+06	2027150	53.10%
14	980340	954550	1068970	1.00E+06	2027150	49.39%
16	1.06E+06	924540	1072680	1.02E+06	2027150	50.34%
18	811460	798370	810410	8.07E+05	2027150	39.80%
20	997010	766190	884180	8.82E+05	2027150	43.53%
22		721330	805360	7.63E+05	2027150	37.66%
24		714760	618330	6.67E+05	2027150	32.88%
26		693230	638260	6.66E+05	2027150	32.84%
28		694830	659960	6.77E+05	2027150	33.42%

OBP4 binding with E2D

E2D con.	Rep 1	Rep 2	Rep 3	Mean		%
0	742850	573180	734370	683466.7	683466.7	100.00%
2	684850	544540	656670	628686.7	683466.7	91.98%
4	656780	504800	629160	596913.3	683466.7	87.34%
6	637910	481500	604790	574733.3	683466.7	84.09%
8	608910	471360	589460	556576.7	683466.7	81.43%
10	600420	438130	579340	539296.7	683466.7	78.91%
12	563200	426320	534310	507943.3	683466.7	74.32%
14	549140	366560	533740	483146.7	683466.7	70.69%
16	536100	345850	490220	457390	683466.7	66.92%
18	489840	346390	487030	441086.7	683466.7	64.54%
20	463230	321960	456080	413756.7	683466.7	60.54%
22	463690	322540	443890	410040	683466.7	59.99%
24	420990	287870	430060	379640	683466.7	55.55%
26	530800	269130	422730	407553.3	683466.7	59.63%
28		266580	412920	339750	683466.7	49.71%
30		252130	381850	316990	683466.7	46.38%
32		243530	373240	308385	683466.7	45.12%
34		207850	365820	286835	683466.7	41.97%
36		169930	344870	257400	683466.7	37.66%
38		182760	337740	260250	683466.7	38.08%
